# Supplementary material for: Heavy Metal Bioaccumulation in Peruvian Food and Medicinal Products
Source: Foods. 2024 Feb 29;13(5):762. doi: 10.3390/foods13050762 (PMC10931273; doi:10.3390/foods13050762)
Supplement: Supplementary file 1 [file foods-13-00762-s001.zip › Figure SI 4 c.pdf]

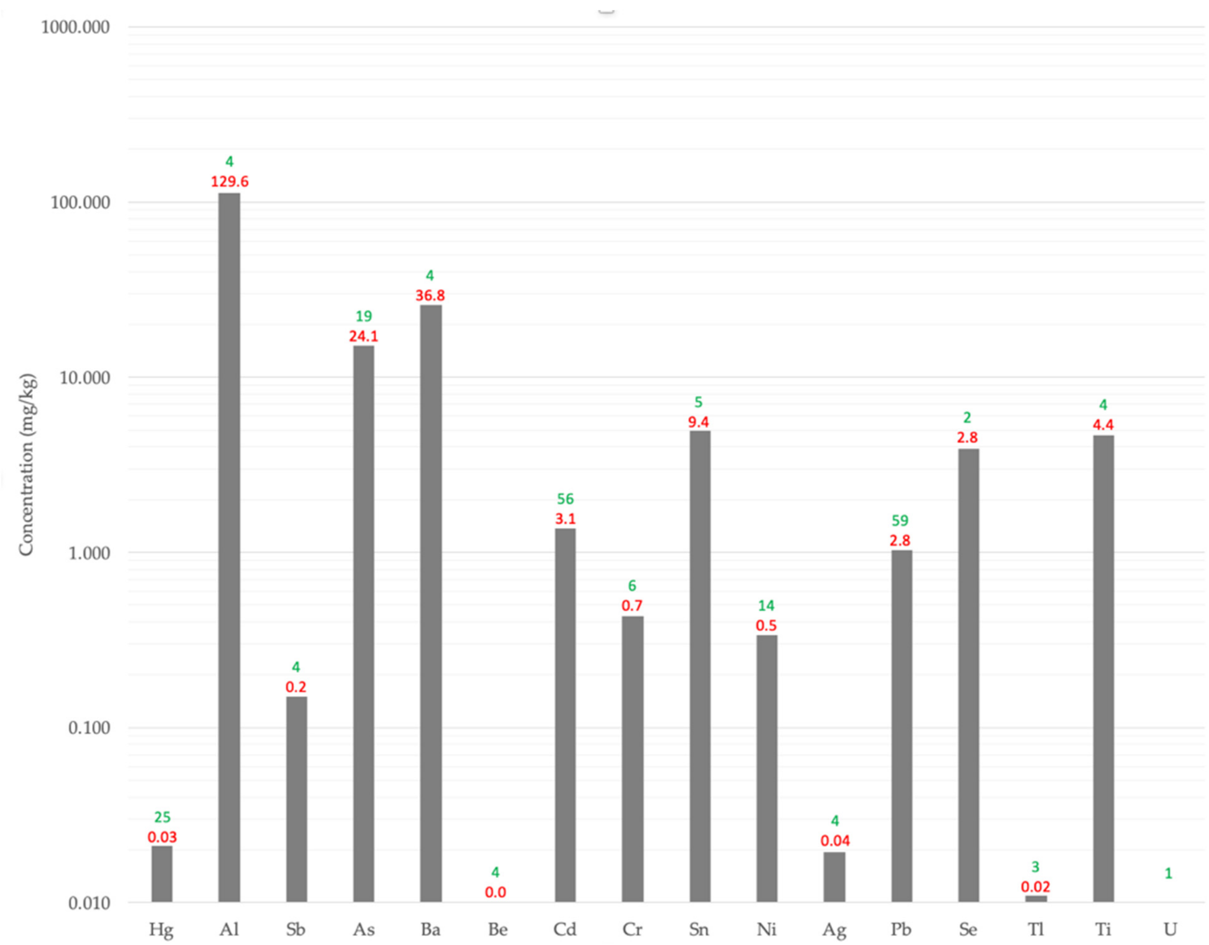

Figure S4. Mean of each metal documented in Peruvian macroinvertebrates and mollusks. Green numbers indicate the quantity of analyses that considered a given metal. WHO standards aren't displayed because they are specific to the food/medicinal type.
