# Supplementary material for: Heavy Metal Bioaccumulation in Peruvian Food and Medicinal Products
Source: Foods. 2024 Feb 29;13(5):762. doi: 10.3390/foods13050762 (PMC10931273; doi:10.3390/foods13050762)
Supplement: Supplementary file 1 [file foods-13-00762-s001.zip › Table SI 1b.pdf]

Table S1. Scientific literature published in English journals (up to 2022).

Decade	Title	Source
1997	Arsenic and heavy metal contamination of soil and vegetation around a copper mine in Northern Peru	Bech et al. [164]
2002	Heavy metal concentrations in fish from a pristine rainforest valley in Peru: a baseline study before the start of oil drilling activities	Gitleb et al. [8]
2012	Shoot accumulation of several trace elements in native plant species from contaminated soils in the Peruvian Andes	Bech et al. [165]
2012	Elevated mercury concentrations in humans of Madre de Dios, Peru	Ashe [166]
2012	A phytoremediation approach using Calamagrostis ligulata and Juncus imbricatus in Andean wetlands of Peru	Alvarez et al. [167]
2014	Metal leaching, acidity, and altitude confine benthic macroinvertebrate community composition in Andean streams	Loayza-Muro et al. [168]
2015	Tissue-specific Cd and Pb accumulation in Peruvian scallop (Argopecten purpuratus) transplanted to a suspended and bottom culture at Sechura Bay, Peru	Loaiza et al. [169]
2015	Mercury Contamination in an Indicator Fish Species from Andean Amazonian Rivers Affected by Petroleum Extraction	Webb et al. [170]
2017	Mercury Levels in Human Hair and Farmed Fish near Artisanal and Small-Scale Gold Mining Communities in the Madre de Dios River Basin, Peru	Langeland et al. [24]
2017	Bioaccumulation of heavy metals in Oncorhynchus mykiss for export at production centers in the Peruvian Central Highlands	Zapata et al. [7]
2017	Mercury concentrations in bats (Chiroptera) from a gold mining area in the Peruvian Amazon	Moreno-Brush et al. [171]
2018	Assessment of polychlorinated biphenyls, organochlorine pesticides, and polybrominated diphenyl ethers in the blood of Humboldt Penguins (Spheniscus humboldti) from the Punta San Juan Marine Protected Area, Peru	Adkesson et al. [172]
2018	Presence of artisanal gold mining predicts mercury bioaccumulation in five genera of bats (Chiroptera)	Kumar et al. [173]
2018	Mercury Contamination in Riverine Sediments and Fish Associated with Artisanal and Small-Scale Gold Mining in Madre de Dios, Peru	Martinez et al. [174]
2018	Potential health risks via consumption of six edible shellfish species collected from Piura – Peru	Loaiza et al. [175]
2018	Accumulation of heavy metals in native Andean plants: potential tools for soil phytoremediation in Ancash (Peru)	Chang-Kee et al. [140]
2019	Potentially toxic metals in lotic systems with aptitude for aquaculture at the watershed Mantaro River, Peru	Custodio et al. [176]
2019	Impact of Heavy Metals on Community Farming Activities in the Central Peruvian Andes	Quispe-Zuniga et al. [177]
2019	Quantitative Determination of Cadmium (Cd) in Soil-Plant System in Potato Cropping (Solanum tuberosum var. Huayro)	Oliva et al. [178]
2019	Cd and Pb reduction in cocoa (Theobroma cacao) nib using two organic amendments	Dávila-Zamora et al. [179]
2019	Predictors of mitochondrial DNA copy number and damage in a mercury-exposed rural Peruvian population near artisanal and small-scale gold mining: An exploratory study	Berky et al. [180]
2019	Efecto del cadmio sobre la mortalidad de adultos de Polydora sp. (Polychaeta: Spionidae) en el laboratorio	Herrera-Perez et al. [27]
2020	Evaluating Wildlife Vulnerability to Mercury Pollution From Artisanal and Small-Scale Gold Mining in Madre de Dios, Peru	Markham and Sangermano [181]
2020	Lead Bioaccumulation in Root and Aerial Part of Natural and Cultivated Pastures in Highly Contaminated Soils in Central Andes of Peru	Berdriñana et al. [182]

2020	Uptake and depletion of the antibiotic sulfadiazine .sup.14C in rainbow trout	Vilca et al. [183]
2020	Monitoring the occurrence of microplastic ingestion in Otariids along the Peruvian and Chilean coasts	Perez-Venegas et al. [184]
2020	Effect of urea on lead absorption in corn (Zea mays L.), spinach (Spinacia oleracea L.) and cabbage (Brassica oleracea L.)	Peñafiel-Sandoval and Iannacone [185]
2020	Soil, Site, and Management Factors Affecting Cadmium Concentrations in Cacao-Growing Soils	Scaccabarozzi et al. [186]
2020	Mercury bioaccumulation in tropical bats from a region of active artisanal and small-scale gold mining	Carrasco-Rueda et al. [187]
2020	Cadmium Uptake in Native Cacao Trees in Agricultural Lands of Bagua, Peru	Oliva et al. [188]
2020	Study to Determine Levels of Cadmium in Cocoa Crops Applied to Inland Areas of Peru: "The Case of the Campo Verde-Honoria Tournavista Corridor"	Rosales-Huamani et al. [60]
2020	Elevated Hair Mercury Levels Are Associated With Neurodevelopmental Deficits in Children Living Near Artisanal and Small-Scale Gold Mining in Peru	Reuben et al. [189]
2020	Mislabelling and high mercury content hampers the efforts of market-based seafood initiatives in Peru	Biffi et al. [26]
2020	Potentially toxic metals and metalloids in surface water intended for human consumption and other uses in the Mantaro River watershed, Peru	Custodio et al. [190]
2020	Citizen science campaign reveals widespread fallout of contaminated dust from mining activities in the central Peruvian Andes	Molloy et al. [30]
2020	Inexpensive Organic Materials and Their Applications towards Heavy Metal Attenuation in Waters from Southern Peru	Garcia-Chevesich et al. [191]
2020	Treatment of tannery wastewater in a pilot scale hybrid constructed wetland system in Arequipa, Peru	Zapana et al. [192]
2021	Lead transfer in the soil-root-plant system in a highly contaminated Andean area	Castro-Bedriñana et al. [193]
2021	Accumulation of As, Ag, Cd, Cu, Pb, and Zn by Native Plants Growing in Soils Contaminated by Mining Environmental Liabilities in the Peruvian Andes	Cruzado-Tafur et al. [141]
2021	Cadmium, Lead and Zinc in the Soil-Plant-Alpaca System and Potential Health Risk Assessment Associated with the Intake of Alpaca Meat in Huancavelica, Peru	Orellana et al. [138]
2021	Bioconcentration and bioaccumulation of toxic metals in Scirpus californicus from natural wetlands in the Central Andes of Peru	Boleji et al. [194]
2021	A population-based mercury exposure assessment near an artisanal and small-scale gold mining site in the Peruvian Amazon	Weinhouse et al. [25]
2021	Insights from Water Quality of High Andean Springs for Human Consumption in Peru	Choque-Quispe et al. [195]
2021	Human risk associated with the ingestion of artichokes grown in soils irrigated with water contaminated by potentially toxic elements, Junin, Peru	Custodio et al. [196]
2021	Mercury Exposure and Toxicological Consequences in Fish and Fish-Eating Wildlife from Anthropogenic Activity in Latin America	Canham et al. [197]
2022	Lead and Cadmium Bioaccumulation in Fresh Cow's Milk in an Intermediate Area of the Central Andes of Peru and Risk to Human Health	Chirinos-Peinado et al. [4]
2022	Mercury concentrations, biomagnification and isotopic discrimination factors in two seabird species from the Humboldt Current ecosystem	Le Croizier et al. [198]
2022	Cadmium and lead levels in muscle tissue of blue shark (Prionace glauca) in the Southeastern Pacific Waters	Cordero-Maldonado et al. [199]
2022	Amazon forests capture high levels of atmospheric mercury pollution from artisanal gold mining	Gerson et al. [31]
