# Supplementary material for: Heavy Metal Bioaccumulation in Peruvian Food and Medicinal Products
Source: Foods. 2024 Feb 29;13(5):762. doi: 10.3390/foods13050762 (PMC10931273; doi:10.3390/foods13050762)
Supplement: Supplementary file 1 [file foods-13-00762-s001.zip › Table SI 2b.pdf]

**Table S2.** Maximum permissible limits (MPLs) of different food/medicinal types, under the World Health Organization's (Codex Alimentarious) standards ($CH_3Hg$ = methyl mercury).

| Food type | Hg | $CH_3$-Hg | Al | Sb | As | Ba | Be | Cd | Cr | Sn | Ni | Ag | Pb | Se | Tl | Ti | U |
|---|---|---|---|---|---|---|---|---|---|---|---|---|---|---|---|---|---|
| Berries and other small fruits | | | | | | | | | | | | | 0.10 | | | | |
| Canned beverages | | | | | | | | | | 150 | | | | | | | |
| Canned chestnuts and Canned chestnuts puree | | | | | | | | | | | | | 0.05 | | | | |
| Canned foods (other than beverages) | | | | | | | | | | 250 | | | | | | | |
| Cephalopods | | | | | | | | 2.00 | | | | | | | | | |
| Cereal grains | | | | | | | | 0.10 | | | | | 0.20 | | | | |
| Chocolate (>70% cacao) | | | | | | | | 0.90 | | | | | | | | | |
| Chocolate (50-70% cacao) | | | | | | | | 0.80 | | | | | | | | | |
| Corned beef | | | | | | | | | | 50 | | | | | | | |
| Cranberry | | | | | | | | | | | | | 0.20 | | | | |
| Cucumbers (pickled) | | | | | | | | | | | | | 0.10 | | | | |
| Currants | | | | | | | | | | | | | 0.20 | | | | |
| Edible fats and oils | | | | | 0.1 | | | | | | | | 0.1 | | | | |
| Edible offal of cattle | | | | | | | | | | | | | 0.20 | | | | |
| Edible offal of pig | | | | | | | | | | | | | 0.15 | | | | |
| Edible offal of poultry | | | | | | | | | | | | | 0.10 | | | | |
| Elderberry | | | | | | | | | | | | | 0.20 | | | | |
| Fat spreads and blended spreads | | | | | 0.1 | | | | | | | | 0 | | | | |
| Fish | | | | | | | | | | | | | 0.30 | | | | |
| Fish (alfonsino) | | 1.50 | | | | | | | | | | | 0.30 | | | | |
| Fish (marlin) | | 1.70 | | | | | | | | | | | 0.30 | | | | |
| Fish (shark) | | 1.60 | | | | | | | | | | | 0.30 | | | | |
| Fish (tuna) | | 1.20 | | | | | | | | | | | 0.30 | | | | |
| Fruits | | | | | | | | | | | | | 0.10 | | | | |
| Fruits (canned) | | | | | | | | | | | | | 0.10 | | | | |
| Ham (cooked cured) | | | | | | | | | | 50 | | | | | | | |
| Infant formula, formula for special medical purposes intended for infants and follow-up formula | | | | | | | | | | | | | 0 | | | | |
| Jams, jellies, and marmalades | | | | | | | | | | | | | 0.4 | | | | |
| Juices (fruit, obtained exclusively from berries and other small fruits) | | | | | | | | | | | | | 0.05 | | | | |
| Juices (fruit) | | | | | | | | | | | | | 0.03 | | | | |
| Juices (grape) | | | | | | | | | | | | | 0.04 | | | | |
| Mango chutney | | | | | | | | | | | | | 0.4 | | | | |
| Marine bivalve mollusks without vicera (except oysters and scallops) | | | | | | | | 2.00 | | | | | | | | | |
| Mashrooms, fresh farmed (common mushrooms (Agaricus bisporous), | | | | | | | | | | | | | 0.30 | | | | |

| | | | | | |
|---|---|---|---|---|---|
| shiitake mushrooms (Lentinula edodes), and oyster mushrooms (Pleurotus ostreatus)) | | | | | |
| Meat (cooked cured chopped) | | | | 50 | |
| Meat (corned beef) | | | | 50 | |
| Meat (luncheon) | | | | 50 | |
| Meat and fat of poultry | | | | | 0.10 |
| Meat of cattle, pig, and sheep | | | | | 0.10 |
| Milk | | | | | 0.02 |
| Milk (secondary products) | | | | | 0.02 |
| Natural mineral waters | 0.001 | 0.01 | 0.003 | | 0.01 |
| Olives (table) | | | | | 0.40 |
| Pork (cooked cured shoulder) | | | | 50 | |
| Pulses | | | 0.10 | | 0.10 |
| Rice, husked | | 0.35 | | | |
| Rice, polished | | 0.20 | 0.40 | | |
| Salt, food grade | 0.1 | 0.50 | 0.50 | | 1.00 |
| Tomatoes (preserved) | | | | | 0.05 |
| Vegetables (brassica) | | | 0.05 | | 0.10 |
| Vegetables (bulb) | | | 0.05 | | 0.10 |
| Vegetables (canned) | | | | | 0.10 |
| Vegetables (fruiting) | | | 0.05 | | 0.05 |
| Vegetables (leafy) | | | 0.02 | | 0.30 |
| Vegetables (legume) | | | 0.10 | | 0.10 |
| Vegetables (root and tuber) | | | 0.10 | | 0.10 |
| Vegetables (stalk and stem) | | | 0.10 | | |
| Wheat | | | 0.20 | | |
| Wine | | | | | 0.10 |
| Wine (fortified / Liqueur) | | | | | 0.15 |
| Wine (wine and fortified / liqueur wine) | | | | | 0.20 |
| Medicinal | | | 0.3 | | 10 |
