# Supplementary material for: Heavy Metal Bioaccumulation in Peruvian Food and Medicinal Products
Source: Foods. 2024 Feb 29;13(5):762. doi: 10.3390/foods13050762 (PMC10931273; doi:10.3390/foods13050762)
Supplement: Supplementary file 1 [file foods-13-00762-s001.zip › Table SI 3b.pdf]

Table S3. Number of publications from each research institution considered in this review, for each food/medicinal category. Unlisted institutions did not have any publications.

Research institution	Plants	Fish	Macroinvertebrates and mollusks	Mammals	Others	Sum
Universidad Nacional de Trujillo	16	2	1		5	24
Universidad Nacional Mayor de San Marcos	13	3		2	2	20
Universidad Nacional del Centro del Perú	14	1		2		17
Universidad Nacional de la Amazonía Peruana	7	2		6		15
Universidad Nacional Agraria de la Selva	11	2				13
Universidad Nacional de San Agustín de Arequipa	5	3	4			12
Universidad Nacional de Ucayali	9	2				11
Universidad Nacional Daniel Alcides Carrión	7	1			1	9
Universidad Nacional Agraria La Molina	5		2		1	8
Universidad Nacional Hermilio Valdizán de Huánuco	4	1			1	6
Universidad Nacional del Altiplano	4	1				5
Universidad Nacional de Piura	5					5
Universidad Nacional Toribio Rodríguez de Mendoza de Amazonas	5					5
Universidad Nacional de Moquegua		1	1	1	2	5
Universidad Nacional Jorge Basadre Grohmann	1	2	1			4
Universidad Nacional de Huancavelica	2	2				4
Universidad Nacional de San Cristóbal de Huamanga	2	1				3
Universidad Nacional de San Antonio Abad del Cusco	2	1				3
Universidad Nacional de San Martín	3					3
Universidad Nacional de Juliaca	3					3

Universidad Nacional de Jaén	3					3
Universidad Nacional San Luis Gonzaga	2					2
Universidad Nacional de Cajamarca	1			1		2
Universidad Nacional Federico Villarreal	2					2
Universidad Nacional Santiago Antúnez de Mayolo	1		1			2
Universidad Nacional del Santa			1	1		2
Universidad Nacional Micaela Bastidas de Apurímac		1			1	2
Revista Chemical Data Collections	2					2
Revista de Investigaciones Veterinarias del Perú		2				2
Universidad Nacional de Ingeniería	1					1
Universidad Nacional del Callao		1				1
Universidad Nacional Amazónica de Madre de Dios	1					1
Revista Scientia Agropecuaria	1					1
Revista Science of The Total Environment	1					1
Revista Forestal del Perú de la Universidad Nacional Agraria La Molina	1					1
Revista Chemosphere	1					1
Revista Avances en Ciencias e Ingeniería	1					1
Organismo de Evaluación y Fiscalizacion Ambiental	1					1
Salud y Tecnología Veterinaria		1				1
Revista Peruana de Biología de la UNMSM		1				1
Revista Perú Biol de Universidad Nacional Mayor de San Marcos			1			1
Revista de Investigación Científica REBIOL		1				1
Revista de Investigación Científica de Universidad Nacional de Juliaca			1			1

Revista Ciencia y Desarrollo de la Universidad Alas Peruanas	1		1
Ministerio del Ambiente	1		1
Instituto Tecnológico de la Producción		1	1
Instituto Tecnológico Pesquero del Perú	1		1
Instituto del Mar del Perú		1	1
